# Role of Supraspinal Neuroinflammation in Chronic Pain After Experimental Spinal Cord Injury—A Systematic Review

**DOI:** 10.3390/ijms27135711

**Published:** 2026-06-24

**Authors:** Telma Ferreira, Célia Duarte Cruz, José Tiago Costa-Pereira

**Affiliations:** 1 Department of Biomedicine, Experimental Biology Unit, Faculty of Medicine of Porto, University of Porto, Alameda Professor Hernani Monteiro, 4200-319 Porto, Portugal; 2 Pain Neurobiology, Instituto de Investigação e Inovação em Saúde-i3S and IBMC, University of Porto, 4200-135 Porto, Portugal; 3 Faculty of Nutrition and Food Sciences, University of Porto, Rua do Campo Alegre 823, 4150-180 Porto, Portugal

**Keywords:** spinal cord injury, supraspinal neuroinflammation, chronic pain, animal models, PRISMA, systematic review

## Abstract

Spinal cord injury (SCI) is a major cause of long-term disability and is frequently accompanied by chronic pain, substantially reducing quality of life. Although spinal neuroinflammation is a recognized contributor to neuropathic pain, the role of supraspinal neuroinflammation remains less well defined. This systematic review critically evaluated experimental evidence linking SCI-induced supraspinal neuroinflammation with pain-related behaviors in animal models. A systematic literature search in PubMed, Web of Science Core Collection, and Scopus identified studies published over the last 20 years using rodent SCI models that assessed both supraspinal neuroinflammatory markers and pain-related behaviors. After screening, nine studies met the predefined criteria. The analyzed studies suggested that SCI is associated with supraspinal neuroinflammatory alterations, including increased microglial and astrocytic activation and upregulation of pro-inflammatory cytokines and chemokine-related pathways, in several brain regions. In intervention studies, reduced neuroinflammation was accompanied by improvement in mechanical or thermal pain-related behaviors. However, considerable methodological heterogeneity and moderate to high risk of bias were observed. Current evidence suggests an association between supraspinal neuroinflammatory alterations and chronic pain-related behaviors after SCI, but the limited number of studies and methodological variability restrict firm conclusions. Further well-designed experimental studies are needed to clarify underlying mechanisms.

## 1. Introduction

Spinal cord injury (SCI) remains one of the most relevant worldwide causes of disability, with a tremendous impact on patients, relatives, caregivers, and society in general. It is an important pressure point for healthcare systems and is accompanied by several comorbidities that greatly degrade the quality of life. Most SCI cases are of traumatic origin, and the leading causes include road traffic injuries, falls and violence. SCI causes major disturbances in sensory, motor and autonomic function, leading to permanent loss of function [1,2].

Besides sensorimotor impairment, chronic pain is one of the most significant and debilitating SCI-induced impairments [3]. In fact, pain, defined by the International Association for the Study of Pain as “An unpleasant sensory and emotional experience associated with, or resembling that associated with, actual or potential tissue damage” [4], is reported at all stages of disease progression. Acute pain arises after trauma and remains present during initial recovery, receding with tissue scarring, whereas chronic pain emerges in time due to maladaptive neuroplasticity. Chronic pain strongly impairs patients’ quality of life and often exceeds the impact of other functional disabilities. Central neuropathic pain (CNP) is reported by more than 50% of SCI patients and reflects SCI-induced damage to the somatosensory system, more specifically the spinal cord and/or the brain [5]. Pain may be spontaneous or evoked by peripheral stimulation [6,7] and course with allodynia and/or hyperalgesia [8]. CNP is divided into at-, above- and below-level neuropathic pain, the latter being the most common form of pain in SCI patients [9]. It is typically of central origin, felt diffusely below the level of injury and appears when chronicity has set [10,11].

In spinal intact individuals, pain perception is typically initiated by activation of nociceptors, which are primary afferent sensory fibers, with cell bodies located in the dorsal root ganglia, for the body, and the trigeminal ganglion, for the face [12]. These specialized sensory afferents have long processes that project to the dorsal horn of the spinal cord, where sensory input is conveyed to spinal neurons. From the spinal cord, noxious input is conveyed to supraspinal locations via ascending pain pathways, most notably the spinothalamic, spinoreticular, spinomesencephalic, cervicothalamic, and spinohypothalamic tracts [13]. In the brain, pain processing involves multiple structures, including the thalamus, which operates as a relay hub, the somatosensory cortex, anterior cingulate gyrus and insular cortex [12,14]. Other structures such as the hypothalamus, nucleus tractus solitarius, rostroventromedial medulla, parabrachial nucleus, periaqueductal gray and cortico-limbic system play key roles in the affective and cognitive dimensions of pain [15]. A descending response is then conveyed back to the spinal cord via modulatory pathways, which may exert inhibitory or facilitatory effects. While not anatomically separated in the brain, inhibitory influences mainly descend via the dorsolateral funiculi, whereas facilitatory influences predominantly reach the spinal cord through the ventrolateral funiculi [16,17,18]. Given the complexity of ascending and descending nociceptive neurotransmission, described in detail elsewhere [19,20], it comes as no surprise that SCI significantly compromises these pathways and undermines intrinsic pain regulation mechanisms, critically leading to CNP.

Another key mechanism contributing to CNP is neuroinflammation, characterized by activation of glial cells, including microglia, astrocytes and oligodendrocytes in the brain and spinal cord [21,22]. Activation of these cells is induced by SCI and is accompanied by migration of peripheral immune cells, such as neutrophils, monocytes and B- and T- lymphocytes [23,24], as the blood–spinal cord barrier is disrupted by spinal tissue damage. This intense inflammatory response is important for tissue repairing at the lesion site, but neuroinflammation is often neurotoxic, fails to clear necrotic debris and is not an effective initiator of healing [25]. Glial cells and migrating immune cells acquire a pro-inflammatory phenotype, with significant morphological changes such as cellular hypertrophy and process retraction or extension and increased expression of microglial and astrocytic markers, such as CD11b, IBA1, CX3CR1 (microglial markers) and GFAP (a marker of the astrocyte population) [26,27]. Synthesis of interleukins and other inflammatory mediators is also upregulated [23]. Accordingly, in animal models of SCI, it has been demonstrated that spinal trauma is followed by upregulation of spinal levels of the interleukins TNF-α [28,29], IL-1β [30] and the neurotrophin NGF [31,32], a powerful regulator of neuronal plasticity in the context of pain [33]. While the importance of neuroinflammation at the spinal cord is widely recognized as an important mechanism in CNP, neuroinflammation is not restricted to the spinal cord but is also evident in supraspinal regions of the nervous system [34,35]. It is, however, less clear if supraspinal neuroinflammation is a key component in the development and maintenance of chronic pain. To address this question, a systematic review was conducted following the PRISMA guidelines to critically evaluate available data describing neuroinflammation levels in supraspinal nuclei involved in pain processing and the effects of interventions to harness inflammation.

## 2. Materials and Methods

### 2.1. Literature Search

This systematic review was conducted following the PRISMA 2020 checklist [36], aiming to identify and analyze scientific articles of experimental spinal cord injury models with supraspinal neuroinflammation and behavioral or physiological measures of pain. It is registered on the PROSPERO database for systematic reviews, with the number CRD420251162389 and can be accessed via: https://www.crd.york.ac.uk/PROSPERO/view/CRD420251162389 (accessed on 28 April 2026). On 21 July 2025, we searched in three databases: PubMed (via PubMed), Web of Science Core Collection (via Web of Science) and Scopus (via Scopus). The search was limited to articles published between 1 January 2005 and 21 July 2025. For PubMed, the following query was used: (SCI OR “spinal cord injur*” OR “spinal cord-injur*”) AND (brain* OR supraspina* OR central* OR “central nervous system”) AND (neuroinflamma* OR inflamma* OR interleuk* OR cytokine* OR chemokine*) AND (pain OR nocicept* OR hypersensitivi* OR hyperalges* OR allodynia) AND (rat OR rats OR mice OR mouse OR rodent*), and 748 articles were found. In Web of Science, the query used was: TS=(SCI OR “spinal cord injur*” OR “spinal cord-injur*”) AND TS=(brain* OR supraspina* OR central* OR “central nervous system”) AND TS=(neuroinflamma* OR inflamma* OR interleuk* OR cytokine* OR chemokine*) AND TS=(pain OR nocicept* OR hypersensitivi* OR hyperalges* OR allodynia) AND TS=(rat OR rats OR mice OR mouse OR rodent*). Two hundred and sixty-four publications were obtained. The following query was used in Scopus: TITLE-ABS-KEY (SCI OR “spinal cord injur*” OR “spinal cord-injur*”) AND TITLE-ABS-KEY (brain* OR supraspina* OR central* OR “central nervous system”) AND TITLE-ABS-KEY (neuroinflamma* OR inflamma* OR interleuk* OR cytokine* OR chemokine*) AND TITLE-ABS-KEY (pain OR nocicept* OR hypersensitivi* OR hyperalges* OR allodynia) AND TITLE-ABS-KEY (rat OR rats OR mice OR mouse OR rodent*). Two hundred and seventeen publications were retrieved. No filters were used in the PubMed search, while in Scopus and Web of Science, the filter “article” was utilized.

### 2.2. Selection

The studies retrieved were imported to EndNote, and duplicated articles were excluded. The resultant articles, 966, were submitted to title and abstract screening, independently conducted by two investigators. The inclusion criteria used were: articles written in English; full-text access; experimental studies with rodent SCI models, with appropriate control or sham group; evaluation of inflammatory markers, mediators or cellular responses in brain or brainstem regions (supraspinal regions); and pain-related behavioral assessment with and without therapeutic intervention. The exclusion criteria used were: human studies; in vitro studies or experimental studies with animals other than rodents; studies without evaluation of inflammatory markers, mediators or cellular responses in brain regions; studies without pain-related behavioral assessment; studies without appropriate control or sham group; and studies without indication of the timepoint of assessment. The articles that satisfied all exclusion and inclusion criteria were sent for full-text screening. The risk of bias was assessed independently by two working reviewers using an adaptation of the SYRCLE’S risk of bias tool for experimental SCI studies [37]. This was necessary, as the available tool does not address the specific characteristics of pre-clinical SCI research. This adaptation is provided as Appendix A (Table A1). Discrepancies between reviewers were discussed and resolved by consensus following discussion with all authors.

### 2.3. Data Extraction

Two independently working reviewers extracted data for the following outcomes: study characteristics (author and year of publication); animal species, strain, sex, age used; method used to produce the injury and parameter used to evaluate the severity of injury; test, time, frequency and region used for pain assessment; analgesia used; brain region, marker, cellular mechanism and method of quantification of neuroinflammation and intervention targeting neuroinflammation (doses, method, timing and observed effect). If any of these study characteristics were not evident from full-text analysis, they are described as unmentioned. The data is included in Table 1.

## 3. Results

### 3.1. Data Collection

**Figure 1:**
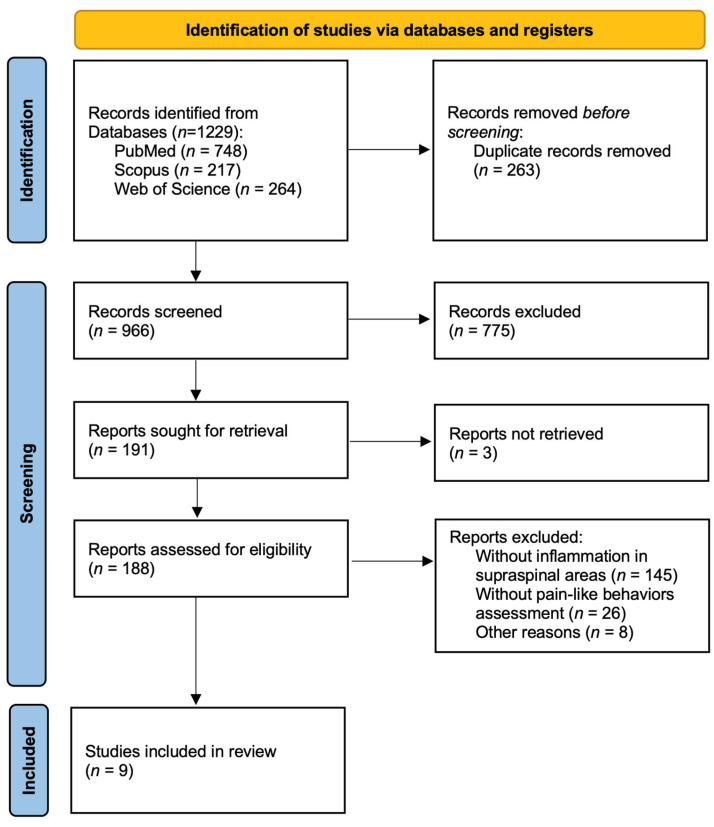
PRISMA flow diagram illustrating the study identification and selection process for this systematic review.

The literature search was conducted on 21 July 2025 in PubMed, Web of Science Core Collection, and Scopus, yielding a total of 1229 records (748, 217, and 264, respectively). After removal of 263 duplicate entries, 966 articles remained for title and abstract screening. Following this initial screening, 775 records were excluded for not meeting the predefined inclusion criteria, and three reports could not be retrieved. The remaining 188 articles underwent full-text assessment for eligibility. Of these, 179 were excluded after detailed evaluation. The most frequent reason for exclusion at this stage was the absence of evidence of supraspinal inflammatory changes (*n* = 145), followed by lack of assessment of pain-related behaviors (*n* = 26). An additional eight studies were excluded for other methodological reasons. Ultimately, nine studies fulfilled all eligibility criteria and were included in the present systematic review. Inter-rater agreement for the study selection phase was very high (Kappa coefficient: 0.86). The study selection process is illustrated in the PRISMA flow diagram (Figure 1).

### 3.2. Characterization of the Animal Model

From the analysis of the included studies, data regarding animal characteristics, namely species, strain, sex, age, and weight, were extracted. Most studies were conducted in rats (*n* = 6/9), while the remaining studies used mouse models (*n* = 3/9), all of which chose the ICR-CD1 strain. In rat studies, the Sprague–Dawley strain was predominantly used (*n* = 5/9), whereas one study employed the Wistar strain (*n* = 1/9). All mouse studies were conducted in female animals (*n* = 3/9). In rat studies, the use of male and female animals was equally distributed, with three studies using males and three using females.

The reporting of animal age was heterogeneous across studies. Qualitative descriptors such as “adult” were used in two mouse studies (*n* = 2/9) and in one rat study (*n* = 1/9), while the term “young adult” was used in one rat study (*n* = 1/9). However, three rat studies failed to report the specific animal age (n = 3/9). Only two studies (n = 2/9) provided specific age information: one using mice with five-week-old animals and one using rats with 90 to 110 days old. In contrast, body weight was reported in all included studies.

### 3.3. Induction of SCI

The studies included in this review used models of spinal cord injury at thoracic levels, with lesions performed between thoracic levels T8 and T12. The most frequently used method to induce SCI was contusion (*n* = 7/9), followed by compression injuries (*n* = 2/9). All mouse studies employed a weight-drop contusion model at the T8–T9 level (*n* = 3/9), resulting exclusively in mild injuries. In rat studies, contusion injuries were induced using an impactor in four studies (*n* = 4/9). Compression injuries were less frequently used, with one study employing a clip (*n* = 1/9) and another using an impounder device (*n* = 1/9). In what refers to the severity of injury, four rat studies did not report the parameters used to define lesion severity (*n* = 4/9). One study induced a moderate injury (*n* = 1/9), while another included multiple experimental groups with mild, moderate, and severe injuries (*n* = 1/9). In contrast, all mouse studies reported the induction of mild SCI (*n* = 3/9).

### 3.4. Pain Assessment Methods

All included studies used reflexive behavioral tests to assess pain-related behaviors. One study additionally incorporated non-reflexive measures by evaluating spontaneous pain. Most studies employed both thermal and mechanical sensitivity tests (*n* = 5/9), whereas two studies assessed mechanical sensitivity only (*n* = 2/9) and two others exclusively evaluated thermal sensitivity (*n* = 2/9). Additional behavioral assessments were performed in six of the nine studies and included tests such as the open-field test, light–dark box, forced swimming test, social interaction test, burrowing test, sucrose preference test, and evaluation of neurological reflexes. One-third of the articles did not report the time of day chosen for behavioral testing. The remaining studies provided this information and indicated that tests were conducted during the light cycle.

All studies assessed pain behavior at multiple time points. The plantar surface of the hindpaw was the most commonly tested anatomical site (*n* = 8/9), while one study referred only to the use of the “rat’s paw” without further specification. The tail sensitivity was tested in one study, and another included the forepaw and the dorsal skin corresponding to the T11–T12 dermatomes.

### 3.5. Mechanisms of Neuroinflammation and Tissue Sites Under Studies

The included studies were also analyzed to identify neuroinflammatory mechanisms, the supraspinal tissue sites examined, and the methodological approaches used. Immunohistochemistry was the most frequently employed technique, described in seven of the nine studies, often in combination with other methods. Western blot analysis was performed in four articles, while quantitative real-time PCR was used in two. Enzyme-linked immunosorbent assay and magnetic bead–based immunoassays were each used in one study.

The studies under our evaluation focused on several supraspinal structures. The presence of signs of neuroinflammation was sought in limbic and prefrontal regions, which were investigated in seven of the nine studies and included the anterior cingulate cortex, medial prefrontal cortex, amygdala, basolateral amygdala, hippocampus, and nucleus accumbens. Brainstem and midbrain regions were examined in four articles, specifically the periaqueductal gray, rostroventromedial medulla, raphe magnus nucleus, parabrachial nucleus, ventral tegmental area, and granular nucleus. Thalamic regions were assessed in three studies and comprised the ventral posterolateral, ventral posteromedial, posterior, and centrolateral nuclei of the thalamus. Cortical sensory areas were less frequently evaluated, being reported in two articles and including the sensory cortex and non-specified cerebral cortex.

With respect to the neuroinflammatory mechanisms investigated, eight of the nine studies evaluated glial reactivity. Activation levels of microglia and/or astrocytes were confirmed by analysis of specific markers, such as IBA1, GFAP, OX42, CD86, CD206 and p38 MAPK. Neuroinflammatory mediators and associated pathways, including pro- and anti-inflammatory cytokines, chemokines, their receptors, and transcriptional regulators involved in inflammatory signaling, were analyzed in seven studies.

Overall, the analyzed studies consistently demonstrated that spinal cord injury induces mechanical allodynia and thermal hyperalgesia, frequently accompanied by supraspinal neuroinflammatory alterations. Glial reactivity was reported in the majority of the studies, with increased GFAP and IBA1 expression observed across cortical, thalamic, and brainstem regions. Several articles also described upregulation of pro-inflammatory cytokines and chemokine-related signaling pathways, particularly involving the CX3CL1/CX3CR1 axis. In some studies, changes in microglial phenotype were reported, consistent with a predominantly pro-inflammatory profile following SCI. However, these neuroinflammatory alterations were not uniformly distributed, as certain brain regions did not exhibit significant changes.

### 3.6. Studies Interventions

Among the selected studies, three did not include any intervention targeting neuroinflammation. The remaining studies (*n* = 6/9) evaluated interventions aimed at modulating neuroinflammatory processes. Most of the interventional studies employed systemic administration of pharmacological compounds (*n* = 5/9), including polyphenolic extracts derived from grape stalks and coffee, docosahexaenoic acid, L-arginine, deferoxamine, minocycline, and the cyclin-dependent kinase inhibitor CR8. These compounds were administered either intraperitoneally or intravenously and followed acute or repeated dosing regimens, depending on the study design. In contrast, one study investigated a non-pharmacological neuromodulatory approach, consisting of transcranial direct current stimulation applied daily through stereotaxically implanted cortical electrodes. Details regarding dosing, timing, routes of administration, and treatment duration for each intervention are summarized in Table 1.

Across the interventional studies, attenuation of pain-like behaviors was consistently associated with modulation of supraspinal neuroinflammation. Improvements in mechanical and thermal sensitivity were accompanied by reductions in microglial and astrocytic activation, decreased expression of pro-inflammatory cytokines, and, in some cases, a shift toward a microglial phenotype associated with anti-inflammatory signaling.

### 3.7. Risk of Bias Analysis

**Figure 2:**
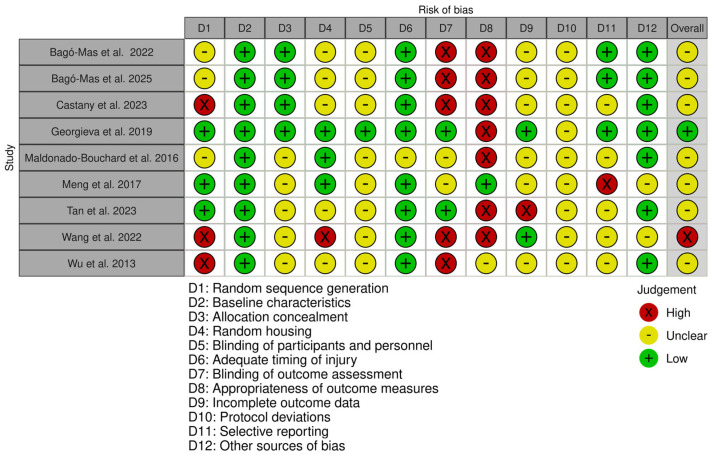
Risk of bias assessment of the included studies using the adapted SCIRCLE tool for spinal cord injury animal models [38,39,40,41,42,43,44,45,46].

The methodological quality of the included studies was assessed using an adapted version of the SYRCLE Risk of Bias tool [37] for animal models of spinal cord injury (available as Table A1), with the results summarized in Figure 2. Overall, the risk of bias across studies was predominantly classified as high or unclear. Only one study achieved a low risk profile, with 10 responses out of 12 rated as low risk of bias, whereas all remaining studies showed a predominance of domains scored as either high or unclear risk. Across all included studies, the domain baseline group similarity was consistently rated as low risk of bias. Additionally, most studies were classified as low risk in the domains’ other sources of bias (*n* = 7/9) and adequacy of injury timing (*n* = 8/9).

On the other end, most studies presented high or unclear risk of bias in several key methodological domains, particularly those related to randomization procedures, allocation concealment, housing and intervention blinding, outcome assessment blinding, appropriateness of outcome measures, management of incomplete outcome data, protocol deviations, and selective outcome reporting.

## 4. Discussion

This systematic review provides a comprehensive overview of the available evidence regarding supraspinal neuroinflammatory alterations associated with the development and maintenance of chronic neuropathic pain following spinal cord injury (SCI). While neuroinflammation at the spinal cord level is well established as a key contributor to neuropathic pain of peripheral [47] and central origin [48], considerably less attention has been given to the importance of the same mechanisms in supraspinal regions involved in pain processing [49,50,51]. This matter was investigated in the present systematic review that focused on experimental data regarding SCI. We found a total of 966 studies, but only 9 were included. The majority of studies were excluded due to the absence of evaluation of inflammatory markers, mediators, or cellular responses in brain regions, and the absence of pain-related behavioral assessment. From the selected studies, only one study presented a low risk of bias, with most studies presenting a moderate to high risk of bias. Our analysis of the risk of bias relied on a tailor-made tool, which adapted the SYRCLE Risk of Bias tool for experimental research. There were several reasons for the high or unclear risk of bias, including lack of randomization, allocation concealment, incomplete data report and/or selective outcome reporting. Other methodological domains with high risk of bias were blinding of the intervention, assessment of the outcomes, appropriateness of outcome measures and deviations to the protocol. While these issues should normally be avoided, it should be remembered that experimental models of SCI are challenging and produce obvious effects on animals undergoing surgical intervention, whether it was a sham procedure or surgical spinal trauma. The effects of experimental SCI and specific post-surgical care, which often require adjustments to the protocol to respect and promote the animals’ welfare, as well as the response to experimental drugs, may preclude full blinding, as the effects might be obvious.

Overall, the analyzed studies indicate that SCI is accompanied by supraspinal neuroinflammatory responses, characterized by increased astrocytic and microglial reactivity, upregulation of pro-inflammatory cytokines and chemokine-related signaling pathways, and, in some cases, changes in microglial marker profiles commonly associated with pro-inflammatory responses [38,39,40,43,44,45,46]. When interpreting these findings, it is important to acknowledge that one of the included studies described microglial phenotypic changes using the traditional M1/M2 terminology, referring to M1-like microglia as predominantly pro-inflammatory and M2-like microglia as predominantly anti-inflammatory [44]. However, this dichotomous classification is currently seen as an oversimplification, as microglial states are increasingly understood as dynamic, context-dependent, and distributed along a functional continuum rather than as two clearly separated phenotypes [52,53,54]. Therefore, in the present review, M1/M2-related terminology was not adopted as a definitive classification. Instead, when referring to that study [44], M1/M2-related terminology was interpreted only as an operational descriptor of the pro- and anti-inflammatory marker profiles reported by the original authors. This should therefore be regarded as a limitation of the present review. Any synthesis of original observations depends on the terminology and marker-based interpretation reported in the original study, which may oversimplify the complexity of microglial responses after SCI. Consistent with a potential role of supraspinal neuroinflammation in SCI-induced pain, interventions that attenuated supraspinal neuroinflammation were associated with improvements in pain-like measures [38,39,41,43,44,46]. Nevertheless, these findings should be interpreted as supporting an association rather than establishing causality.

In this review, most included studies assessed glial reactivity by evaluating the expression of markers of microglial and astrocytic activation, respectively IBA1, GFAP, OX42, CD86, CD206 and p38 MAPK. This strong emphasis likely reflects the well-established role of glial activation in the development of neuropathic pain, where microglia and astrocytes actively contribute to inflammatory signaling [21,27]. Likewise, the majority of studies also evaluated neuroinflammatory mediators, including pro-inflammatory cytokines and chemokine-related pathways [38,39,40,42,43,44,45]. This suggests that supraspinal neuroinflammation is likely an interconnected system, in which activated glial cells produce pro-inflammatory mediators that, in turn, further potentiate gliosis, perpetuating neuroinflammation and facilitating persistent nociceptive activation. However, this predominant focus on immunohistochemical markers and inflammatory pathways may prevent a comprehensive understanding of supraspinal mechanisms. None of the included studies directly explored neuronal functional alterations, synaptic plasticity, or connectivity changes within pain-related brain circuits. We propose that a more integrative approach combining inflammatory, neuronal, and circuit-level analyses is necessary to fully elucidate the contribution of supraspinal neuroinflammation to chronic pain after SCI.

Regarding the supraspinal regions analyzed, considerable anatomical heterogeneity was observed. Although the total number of included articles was limited, a wide range of brain areas was investigated, such as anterior cingulate cortex, medial pre-frontal cortex, amygdala, basolateral amygdala, hippocampus, nucleus accumbens, periaqueductal gray, rostroventromedial medulla, raphe magnus nucleus, parabrachial nucleus, ventral tegmental area, granular nucleus, ventral posterolateral, ventral posteromedial, posterior, centrolateral nuclei of the thalamus and sensory cortex [38,39,40,41,42,43,44,45,46]. This regional diversity provides an initial general overview of widespread supraspinal neuroinflammation involvement following SCI. However, it should be noted that variability in anatomical focus may limit direct comparison between studies and hinder the establishment of a regional pattern of neuroinflammatory events. The absence of a standardized experimental framework also contributes to this heterogeneity, making it difficult to determine whether certain regions play a more prominent or consistent role in chronic pain maintenance after SCI or whether reported alterations reflect region-specific mechanisms studied in isolation.

Most studies employed pharmacological interventions, where drugs were administered systemically to modulate neuroinflammatory pathways and alleviate pain-like behaviors. In those studies, an attenuation of mechanical allodynia and thermal hyperalgesia, accompanied by reductions in glial activation and pro-inflammatory mediators, was observed [38,39,41,43,44,46], which further supports a potential link between supraspinal neuroinflammation and chronic neuropathic pain following SCI. However, the evidence summarized in this review should not be interpreted as establishing a causal relationship. Most included studies reported parallel changes in pain-like behaviors and supraspinal neuroinflammatory markers but were not designed to determine whether these inflammatory alterations directly drive pain development or maintenance. Although intervention studies provide some supportive evidence, their number was limited, and most interventions were administered systemically rather than selectively targeting supraspinal regions. Therefore, it remains difficult to confirm whether the observed behavioral improvements resulted from direct modulation of supraspinal mechanisms or from broader systemic or spinal effects. Furthermore, the lack of comparative studies between different therapeutic strategies limits understanding of the relative contribution of distinct inflammatory pathways.

Most included studies used female animals, as during spinal shock, abdominal compression for urine removal is easier in these animals than in males. This is an important aspect, as SCI is more prevalent in the male population [55], but females are generally more likely to develop chronic pain [56]. Even though this increased vulnerability to chronic pain has not been consistently demonstrated in SCI-specific animal populations [57], the predominance of female models may influence interpretation and translational application of experimental research. Experimental studies have also demonstrated sex-dependent differences in microglial activation, cytokine signaling, and neuroimmune interactions within pain-related pathways, indicating that males and females may rely on partially distinct mechanisms during the development and maintenance of chronic pain [58,59,60]. Although sex-dependent neuroimmune mechanisms have been increasingly recognized in pain research, they remain insufficiently characterized in the specific context of SCI-related supraspinal neuroinflammation. Therefore, the predominance of female animals in the available literature on pre-clinical SCI research may limit the generalizability of the findings and the identification of potentially relevant sex-specific mechanisms. Consequently, future studies should incorporate both sexes to clarify sex-dependent similarities and differences in SCI-induced neuroinflammatory events leading to chronic neuropathic pain.

Most studies assessed pain using reflex-based measures, such as mechanical withdrawal thresholds or thermal sensitivity tests [38,39,40,41,42,43,44,45,46]. Although these tests are widely used in experimental pain research and are useful for detecting sensory hypersensitivity, including mechanical allodynia and thermal hyperalgesia, they provide only a partial representation of chronic pain. Reflexive withdrawal responses largely reflect stimulus-evoked sensory processing and may be mediated predominantly by spinal or brainstem circuits, rather than capturing the sensory, affective, social and cognitive dimensions of pain [61,62,63]. This is particularly relevant in the context of the present review, as supraspinal regions are strongly involved in the affective, social and cognitive processing of pain [64]. Therefore, reliance on reflexive tests may limit the ability to establish a direct relationship between supraspinal neuroinflammatory alterations and the broader clinical experience of chronic pain after SCI [4,64]. In addition, most behavioral tests require preserved motor responses, which may be impaired in SCI animals and may confound the interpretation of withdrawal-based outcomes [62]. The limited use of more complex behavioral approaches, such as tests evaluating spontaneous pain, anxiety- or depression-like behaviors, social interaction, reward-seeking behavior, or other affective and motivational domains, restricts the translational relevance of the available evidence. Future studies should therefore combine reflexive measures with non-reflexive and multidimensional behavioral assessments better suited to capture the complexity of chronic pain and its supraspinal components.

An important limitation relates to the search strategy used in this review. The search terms were intentionally designed to address the specific research question of this review, namely, the relation between supraspinal neuroinflammatory alterations and pain-related outcomes following SCI. Consequently, studies focusing on mechanisms, such as glial activation, transcriptomic changes, neuroimmune signaling, or neuroimaging findings, may not have been retrieved if these concepts were not explicitly described using the selected inflammatory and pain-related terminology. While this approach improved the specificity of study identification, it may have reduced sensitivity and contributed to the exclusion of studies investigating broader aspects of supraspinal responses to SCI. Future reviews may benefit from broader search strategies aimed at characterizing supraspinal alterations after SCI beyond classical neuroinflammatory markers.

Overall, the studies included in this review support an association between supraspinal neuroinflammatory alterations and chronic neuropathic pain-related behaviors after SCI. However, the available evidence remains insufficient to establish whether these supraspinal neuroinflammatory changes play a causal role in the establishment or maintenance of chronic pain. This uncertainty is reinforced by the considerable risk of bias, the limited number of included studies, and the substantial heterogeneity across experimental designs, including animal species and sex, SCI models and lesion severity, supraspinal regions analyzed, neuroinflammatory markers, behavioral outcomes, and intervention strategies. This heterogeneity makes direct comparisons between studies difficult and reduces the ability to identify consistent mechanistic patterns across the available evidence. Moreover, it may also reduce reproducibility and translational applicability, as findings obtained in specific models, sexes, brain regions, or behavioral paradigms may not be directly generalizable to other experimental or clinical contexts. Therefore, findings should be interpreted with caution. Further unbiased investigation is needed to fully identify specific neuroinflammatory events operating in perpetuation of post-SCI maintenance of chronic neuropathic pain after SCI, pinpointing such mechanisms as a potential therapeutic target.

## 5. Conclusions

Spinal cord injury has a significant impact on patients and society, not only due to sensorimotor impairment but also because of the high prevalence of chronic neuropathic pain, which significantly compromises patients’ quality of life. The present systematic review provides an overview of the available experimental evidence linking SCI with supraspinal neuroinflammatory alterations associated with pain-related behaviors in animal models. Overall, the analyzed studies suggest that SCI is accompanied by supraspinal neuroinflammatory responses, including increased astrocytic and microglial reactivity and upregulation of pro-inflammatory cytokines and chemokine-related signaling pathways. Importantly, interventions aimed at modulating neuroinflammation were associated with improvements in pain-associated behaviors, supporting a potential association between supraspinal inflammatory mechanisms and SCI-induced chronic pain-related behaviors. However, the strength of these conclusions is limited by the small number of available studies, substantial methodological heterogeneity, predominance of correlational evidence, limited number of selectively supraspinal interventions, and the overall moderate to high risk of bias. Therefore, although current evidence suggests an association between supraspinal neuroinflammatory alterations and chronic pain-related behaviors after SCI, further well-designed studies are required to clarify the specific mechanisms involved and to determine whether these mechanisms have therapeutic relevance.

## Figures and Tables

**Table 1:** Summary of included studies in the systematic review. The data were extracted and sorted into the following categories: species, strain, sex, age, weight, method of injury, injury parameter, tests used for pain assessment, time of testing, frequency of testing, regions of pain assessment, analgesia used, neuroinflammation marker/mediator used, cellular mechanism, brain region, method of quantification, interventions targeting neuroinflammation, doses used, method implemented, timing of intervention and mention of ARRIVE guidelines. ACC: anterior cingulate cortex; AMG: amygdala; BLA: basolateral amygdala; CCL2: C–C motif chemokine ligand 2; CCL21: C–C motif chemokine ligand 21; CCR2: C–C motif chemokine receptor 2; CD206: cluster of differentiation 206; CD86: cluster of differentiation 86; CE: coffee extract; CE10: 10 mg/kg of coffee extract; CE15: 15 mg/kg of coffee extract; CL: centrolateral nucleus of the thalamus; CR8: selective cyclin dependent kinase inhibitor; CX3CL1: C–X3–C motif chemokine ligand 1; CX3CR1: C–X3–C motif chemokine receptor 1; DFX: deferoxamine; DHA: docosahexaenoic acid; EGF: epidermal growth factor; ELISA: enzyme-linked immunosorbent assay; G-CSF: granulocyte-colony stimulating factor; GFAP: glial fibrillary acidic protein; GM-CSF: granulocyte-macrophage colony stimulating factor; GrN: granular nucleus; GRO-KC: growth-regulated keratinocyte chemoattractant; GSE: grape stalks extract; GSE10: 10 mg/kg of grape stalks extract; GSE15: 15 mg/kg of grape stalks extract; GSE20: 20 mg/kg of grape stalks extract; IBA1: ionized calcium-binding adaptor molecule 1; IFN-γ: interferon gamma; IL-1α: interleukin-1 alpha; IL-1β: interleukin-1 beta; IL-2: interleukin-2; IL-4: interleukin-4; IL-5: interleukin-5; IL-6: interleukin-6; IL-10: interleukin-10; IL-12p70: interleukin-12 (p70 subunit); IL-13: interleukin-13; IL-17a: interleukin-17A; IL-18: interleukin-18; iNOS: inducible nitric oxide synthase; IP-10: interferon gamma-induced protein 10; I.P.: intraperitoneal; I.V.: intravenous; LIX: lipopolysaccharide-induced CXC chemokine; MCP-1: monocyte chemoattractant protein-1; MIP-1α: macrophage inflammatory protein-1 alpha; MIP-2: macrophage inflammatory protein-2; mPFC: medial prefrontal cortex; N.A.: not applicable; N.I.: not indicated; NAc: nucleus accumbens; NF-κB: nuclear factor kappa-light-chain-enhancer of activated B cells; OX42: antibody recognizing cluster of differentiation 11b; PA: pain assessment; PAG: periaqueductal gray; PBN: parabrachial nucleus; PO: posterior thalamic nucleus; pp-38 MAPK: phosphorylated p38 mitogen-activated protein kinase; qRT-PCR: quantitative reverse transcription polymerase chain reaction; RANTES: regulated on activation, normal T cell expressed and secreted; RMg: raphe magnus nucleus; RVM: rostroventromedial medulla; SCI: spinal cord injury; T8: eighth thoracic vertebra; T9: ninth thoracic vertebra; T10: tenth thoracic vertebra; T11: eleventh thoracic vertebra; T12: twelfth thoracic vertebra; tDCS: transcranial direct current stimulation; TNF-α: tumor necrosis factor alpha; VEGF: vascular endothelial growth factor; VPL: ventral posterolateral nucleus of the thalamus; VPM: ventral posteromedial nucleus of the thalamus; VTA: ventral tegmental area.

Study	Bagó-Mas et al. 2025 [38]	Bagó-Mas et al. 2022 [39]	Castany et al. 2023 [40]	Georgieva et al. 2019 [41]	Maldonado-Bouchard et al. 2016 [42]	Meng et al. 2017 [43]	Tan et al. 2023 [44]	Wang et al. 2022 [45]	Wu et al. 2013 [46]
Species	Mice	Mice	Mice	Rats	Rats	Rats	Rats	Rats	Rats
Strain	ICR-CD1	ICR-CD1	ICR-CD1	Wistar	Sprague–Dawley	Sprague–Dawley	Sprague–Dawley	Sprague–Dawley	Sprague–Dawley
Sex	Female	Female	Female	Male	Male	Female	Female	Female	Male
Age	Adult	Adult	Five-week-old	N.I.	90–110 days old	N.I.	N.I.	Young adult	Adult
Weight	20–25 g	20–30 g	19–26 g	180–200 g	300–350 g	230 g	230–250 g	230–250 g	275–325 g
Method of injury	Contusion-weight dropping (T8–T9)	Contusion-weight dropping (T8–T9)	Contusion-weight dropping (T8–T9)	Contusion-impactor (T9–T10)	Contusion-impactor (T12)	Contusion-impactor (T9–T10)	Clip-compression (T9–T10)	Compression (T10)	Contusion-impactor (T10)
Injury parameter	Mild	Mild	Mild	N.I.	Mild, Moderate and Severe	N.I.	N.I.	Moderate	N.I.
Tests	Reflexive tests: Thermal (hot) and Mechanical	Reflexive tests: Thermal (hot) and Mechanical	Reflexive tests: Thermal (hot) and Mechanical	Reflexive tests: Mechanical sensitivity (below- and at-injury level)	Reflexive tests: Mechanical sensitivity (below- and at-injury level)	Reflexive tests: Thermal (hot) sensitivity. Non-reflexive: Spontaneous pain	Reflexive tests: Thermal (hot) sensitivity	Reflexive tests: Mechanical and thermal (hot) sensitivity	Reflexive tests: Mechanical sensitivity
Other tests	Open-field, Light–Dark Box test, Forced Swimming test, and Social Interaction test	N.A.	Light–Dark Box test, Social Interaction test and Reward-seeking Behavior test	Burrowing test, Open Field test (thigmotactic behavior), and place escape/avoidance paradigm	Open Field test, Sucrose Preference test, Forced Swimming test, and Shock Probe Burying task	N.A.	N.A.	Neurological reflexes	Neurological functional deficits
Time of testing	Light cycle	Light cycle	Light cycle	Light cycle	Light cycle	N.I.	N.I.	Light cycle	N.I.
Frequency	Multiple timepoints	Multiple timepoints	Multiple timepoints	Multiple timepoints	Multiple timepoints	Multiple timepoints	Multiple timepoints	Multiple timepoints	Multiple timepoints
Regions of PA	Hindpaw’s plantar surfaces	Hindpaw’s plantar surfaces	Hindpaw’s plantar surfaces	Midlateral plantar surface of the hindpaw	Tail and plantar surface of the hindpaw	Rats’ paw	Plantar surface of the hindpaw	Plantar surface of the forepaw (mechanical stimulation) and hindpaw (mechanical and thermal stimulation), dorsal skin of the T11–T12 dermatomes (mechanical stimulation)	Plantar surface of the hindpaw
Analgesia	N.I.	N.I.	Buprenorphine (0.3 mg/kg); carprofen (50 mg/kg)	N.I.	N.I.	N.I.	Buprenorphine	N.I.	N.I.
Marker/mediator	Expression of CCL2/CCR2, CX3CL1/CX3CR1, IBA-1 and GFAP	Expression of CX3CL1/CX3CR1, GFAP, IBA1, CD206 and OX42	Expression of IL-1β, TNF-α, IL-6, IBA1, GFAP, CX3CL1 and CX3CR1	IBA1, GFAP, laminin and pp-38 MAPK	Expression of G-CSF, Leptin, Fractalkine, IL-4, EGF, IL-13, IL-10, Eotaxin, GM-CSF, IL-1α, MIP-1α, IL-1β, IL-12p70, IFN-γ, IL-5, IL-2, IL-6, MCP-1, IP-10, GRO-KC, LIX, MIP-2, VEGF, TNF-α, RANTES, IL-17a, IL-18	Expression of NF-κB and IBA1	Expression of the TNF-α, IL-1β, IL-6, IL-10, IBA-1, CD86 and CD206	Expression of the TNF-α, iNOS, GFAP, and IBA1	Expression of the IBA1, and CCL21
Cellular mechanism	Micro- and Astrogliosis; CCL2/CCR2, and CX3CL1/CX3CR1 signaling pathways	Glial cells reactivity; CX3CL1/CX3CR1 signaling pathway	Glial cells reactivity, cytokines expression, and CX3CL1/CX3CR1 signaling pathways	Glial cells activation	Inflammatory pathways	Microglia activation and inflammatory pathways	Microglia reactivity and regulation of pro-inflammatory and anti-inflammatory cytokines	Glia reactivity and production of pro-inflammatory cytokines	Microgliosis
Brain region	ACC, AMG, PAG and RVM	ACC and PAG	mPFC, NAc and AMG	ACC	Hippocampus	Sensory cortex, Hippocampus and Thalamus	Cerebral cortex, Thalamus (VPL), and Midbrain (PAG and VTA)	GrN, PBN, RMg, PAG, Hippocampus, and BLA	PO, VPL, VPM, and CL
Method of quantification	Western blot	Immunohistochemistry; Western blot	qRT-PCR, Western blot, and Immunohistochemistry	Immunohistochemistry	27 cytokine/chemokines were assessed using a magnetic bead immunoassay	Western blot and Immunohistochemistry	ELISA, Immunohistochemistry, and qRT-PCR	Immunohistochemistry	Immunohistochemistry
Pharm/Non-pharm	Polyphenolic extracts from grape stalks (GSE) and coffee (CE)	Polyphenolic extracts from grape stalks (GSE) and coffee (CE)	N.A.	Docosahexaenoic acid (DHA)	N.A.	L-arginine, DFX, and minocycline	Transcranial Direct Current Stimulation (tDCS)	N.A.	CR8
Dose	15 mg/kg (GSE15) and 10 mg/kg (CE10)	10, 15 and 20 mg/kg GSE (GSE10, GSE15 and GSE20) and 10 and 15 mg/kg CE (CE10 and CE15)	N.A.	250 nmol/kg	N.A.	1.5 mg/kg L-arginine, 100 mg/kg DFX, and 45 mg/kg and 22.5 mg/kg minocycline	200 µA	N.A.	1 mg/kg
Method	I.P.	I.P.	N.A.	I.V.	N.A.	I.P.	Stereotaxic implantation of electrodes in the cerebral cortex	N.A.	I.P.
Timing	First, third, and sixth weeks post-surgery	Thirty minutes after SCI and daily during the first week post-injury	N.A.	Acute treatment: 30 min after surgery and thereafter every 3 days lasting for 6 weeks. Delayed regimen: At the beginning of week 4 after surgery and thereafter every 3 days lasting for 4 weeks.	N.A.	L-arginine and DFX: on the day after surgery, followed by once-weekly injections. Minocycline: after surgery repeated over 12 h.	tDCS stimulation: daily with 200 μA in a total of 10 days of tDCS	N.A.	Once daily beginning 3 h post-injury and continuing for 7 days

## Data Availability

No new data were created or analyzed in this study. Data sharing is not applicable to this article.

## Abbreviations

The following abbreviations are used in this manuscript:

SCI	Spinal cord injury
CNP	Central neuropathic pain
CD11b	Cluster of differentiation 11b
IBA1	Ionized calcium-binding adaptor molecule 1
CX3CR1	C–X3–C motif chemokine receptor 1
GFAP	Glial fibrillary acidic protein
TNF-α	Tumor necrosis factor alpha
IL-1β	Interleukin-1 beta
NGF	Nerve growth factor
PRISMA	Preferred reporting items for systematic reviews and meta-analyses
SYRCLE	SYstematic Review Centre for Laboratory animal Experimentation
T8	Eighth thoracic vertebra
T9	Ninth thoracic vertebra
T12	Twelfth thoracic vertebra
OX42	Antibody recognizing cluster of differentiation 11b
CD86	Cluster of differentiation 86
CD206	Cluster of differentiation 206
p38 MAPK	p38 mitogen-activated protein kinase
CX3CL1	C–X3–C motif chemokine ligand 1
CR8	Selective cyclin dependent kinase inhibitor

## Appendix A

**Table A1:** Adapted version of the SYRCLE Risk of Bias tool for animal models of spinal cord injury. For Injury Severity, studies must demonstrate that the severity of the spinal cord injury is comparable between experimental and control animals before any treatment. Differences in injury could fundamentally alter the inflammatory cascade. For Animal Characteristics, studies must verify that age, sex, and weight are balanced. Age is a significant factor in chronic neuroinflammation. For Behavioral Pain Assessment, the person performing the von Frey filament test (for mechanical allodynia) or Hargreaves test (for thermal hyperalgesia) must be blinded to the animal’s group to prevent bias in interpreting subtle behavioral responses. For Molecular/Histological Assessment, the researcher quantifying cell counts (e.g., IBA1 positive microglia) or protein/mRNA expression in the brain must be blinded to the group to prevent observer bias in selection of fields of view or interpretation of results. For Neuroinflammation, clear descriptions of the brain regions assessed (e.g., thalamus, cortex, hippocampus, periaqueductal gray) and the specific markers used for neuroinflammation (e.g., Microglia: IBA1, CD68; Astrocytes: GFAP, S100B; Cytokines: IL-1β, TNF−α) were sought out. The technique must be appropriate (e.g., a simple Western blot of a whole brain region is less precise than immunohistochemistry with quantitative stereology). For Chronic Pain, studies must ensure pain behaviors are measured at time points considered chronic (e.g., >2−4 weeks post-SCI). For Clear, a study must explicitly describe and appropriately execute the procedure. For Unclear, a study has to explicitly state the procedure was not done or the described procedure is clearly flawed/inadequate (e.g., using a non-randomized method). For Unmentioned, a study has to report that the procedure was done but provide insufficient detail to determine if it was done correctly or fail to report on the item entirely.

SCIRCLE Item	Signaling Question for SCI/Neuroinflammation	Risk Judgment (Clear/Unclear/Unmentioned)	Justification/ Information from Paper/Page
**1. Selection Bias:** addresses systematic differences between groups, primarily due to how animals were allocated.			
**Random sequence generation**	Was the allocation sequence adequately generated and applied? (e.g., random number table, computer generator)		
**Baseline characteristics**	Were the groups similar at baseline, or were they adjusted for confounders in the analysis? Specific to SCI: Were baseline characteristics (e.g., age, sex, weight, pre-injury motor function) balanced? Was the injury severity (e.g., contusion force, depth of lesion) reported and similar between groups?		
**Allocation concealment**	Was the allocation to the different groups adequately concealed? (e.g., centralized randomization, coded containers, sealed envelopes)		
**2. Performance Bias:** relates to systematic differences in the care or treatment (other than the intervention under investigation) provided to the subjects.			
**Random housing**	Was the housing of the animals adequately randomized and/or reported? (i.e., were the animals of different groups housed intermingled?)		
**Blinding of caregivers/personnel**	Was the caregiver/investigator administering the treatment or assessing the animals’ health/behavior blinded to the intervention?		
**Adequate timing of injury/treatment**	**Specific to SCI:** Was the timing of the SCI induction and the subsequent neuroinflammation-related intervention (if applicable) clearly defined and equivalent across groups? (e.g., intervention started 1 h or 7 days post-SCI).		
**3. Detection Bias:** arises from systematic differences between groups in how outcomes are determined. This is crucial for both pain behavior and histological/molecular			
**Blinding of outcome assessors**	Was the outcome assessor blinded to the intervention/group allocation? **Specific to Pain:** Was the person conducting behavioral pain tests (e.g., von Frey, thermal) blinded? **Specific to Neuroinflammation:** Was the person counting cells (microglia, astrocytes) or quantifying molecular markers (e.g., cytokines, Western blots) blinded?		
**Appropriateness of Outcome Measures**	**Specific to Pain:** Were appropriate measures of **chronic neuropathic pain** used (e.g., allodynia/hyperalgesia measured long-term)? **Specific to Neuroinflammation:** Were standard and validated methods used for assessing brain neuroinflammation (e.g., **stereological cell counting**, **specific antibody markers for glial activation in brain regions like the cortex, thalamus, or hippocampus**)?		
**4. Attrition Bias:** caused by systematic differences between groups in withdrawals from the study, deviations from the protocol, or missing outcome data.			
**Incomplete outcome data**	Were all animals included in the analysis, or was the number of animals lost to follow-up adequately reported, and was the missing data handled appropriately? **Specific to SCI:** Is the loss of animals due to injury-related mortality accounted for and balanced between groups?		
**Protocol deviations**	Were there any reported protocol deviations that could systematically affect the outcomes of one group over another?		
**5. Reporting Bias:** stems from systematic differences between reported and unreported findings.			
**Selective outcome reporting**	Was the study protocol or a pre-registration available, and did the reported outcomes align with the pre-specified outcomes? **Specific to Neuroinflammation:** Were all measured neuroinflammatory markers and all behavioral pain outcomes reported, regardless of the direction or significance of the result?		
**6. Other Potential Sources of Bias:** any risk of bias not covered by the above domains that could impact the study’s validity.			
**Other bias**	Are there any other concerns? **Specific to SCI Models:** Was the SCI model appropriate for studying chronic pain (e.g., contusion/compression models more relevant than complete transection for chronic pain)? **Specific to Analgesics:** If analgesics were used post-SCI, were they comparable between groups and non-interfering with the neuroinflammation outcomes? **Specific to Stereology:** If histology was used, did the authors explicitly state the use of **unbiased stereological techniques** for cell counting?		
